# Analysis of results of surgical treatment of posttraumatic stiff elbow

**DOI:** 10.4103/0019-5413.40257

**Published:** 2008

**Authors:** Chandrabose Rex, PM Suresh Kumar, Addagalla Srimannarayana, S Chugh, M Ravichandran, DN Harish

**Affiliations:** Rex Ortho Hospital, Poomarket, Mettupalayam Road, Coimbatore, Tamil Nadu, India

**Keywords:** Post traumatic stiff elbow, fractures around elbow, myositis ossification, dislocation elbow

## Abstract

**Background:**

Surgical management of posttraumatic elbow stiffness has been reported with poor outcome following treatment. Sequential release in earlier stages of stiffness yielded much better results. The goal of our study was to assess the outcome in improvement of the range of motion of the elbow after surgical release and to analyze a tailor-made approach according to individual needs to yield good result.

**Materials and Methods:**

A prospective study was conducted in 47 cases of elbow stiffness due to various types of injuries. All the cases were treated with sequential release if there was no progress after adequate supervised conservative management except in unreduced dislocations. All the cases were followed up for a minimum period of 24 months. Overall outcome was rated with the functional scoring system by Mayo Clinic Performance Index.

**Results:**

Twenty-five (44.68%) out of 47 patients had excellent results with a mean preoperative range of motion of 33.9° and postoperative range of motion of 105° with net gain in range of motion of 71.1° (‘*t*’ test value is 19.27, *P* < 0.01). None of the patients had elbow instability. Patients not having heterotopic ossification, who underwent surgery from three to six months post injury had a mean gain of 73.5°. In patients who waited for more than six months had mean gain of 66.8°. However, the results in cases having heterotopic ossification followed a slightly different pattern. In cases where release was performed from three months to six months had mean gain of 77.5°. Cases in which release was performed after six months had gain of 57.1°.

**Conclusions:**

In cases of posttraumatic elbow stiffness after a failed initial conservative treatment, early arthrolysis with sequential surgical soft tissue release yields good result than delayed surgery.

## INTRODUCTION

Posttraumatic elbow stiffness is common following various elbow injuries due to late presentation and inadequate initial treatment. This results in a spectrum of cases from simple elbow stiffness with normal radiological findings to complex fracture dislocations and heterotopic ossification as viewed in X-ray. The recommended waiting time was 12-18 months in treating heterotopic ossification.^1^ The operative technique and results of surgical management remained unclear.^2^ This was attributed to the nature of the surgery with accompanying potential risk to damage the neurovascular bundle that exists in close proximity to the operating region. So surgical release in posttraumatic elbow stiffness was rarely attempted. However, the recent literature favors early surgical release but results obtained from surgical management in different studies were inconsistent regarding the improvement in range of motion and the waiting period for surgery.^3^–^8^ The goal of our study was to assess the outcome in improvement of the range of motion of the elbow after surgical release and to analyze a tailor-made approach according to individual needs.

## MATERIALS AND METHODS

A prospective study was conducted between December 1999 and December 2004 on surgical release of posttraumatic stiff elbows. Forty-seven cases of stiff elbow were analyzed. All patients having elbow stiffness due to various types of injuries like road traffic accidents, domestic fall and native treatment in the form of massage and oil bandage were included in this study. After an initial active physiotherapy for three to four weeks if the patient felt gross functional disturbance because of stiffness (arc of movement was less than 100°), surgery was offered. The study excluded children below 12 years and cases which were post-infective. This study was approved by the institution review board. All enrolled patients consented to participate in the study after explanation of risks and benefits. The final assessment of the range of motion of both flexion and extension of individuals prior to and after undergoing surgical release was done at a minimum period of two years after the surgery. There were 36 males and 11 females in our study, with a mean age of 30.8 years (14-71 years). The mean time interval between injury and surgery was 12.6 months (3-120 months). Surgery was offered at the earliest when there was altered anatomy of the elbow, which needed to be addressed, like in maluniting fracture, nonunion, dislocation and presence of heterotopic ossification without any predisposing systemic illness. A small group of patients with soft tissue injury alone were offered surgery because of no improvement with adequate physiotherapy and with severe stiffness having only a jog of movement. Such recalcitrant stiff elbows were operated. However if patient showed steady improvement in range of motion with time, on conservative measures they were excluded from study. The minimum follow-up is two years and a maximum of five years, with a mean of 33.9 months. All 47 cases of elbow stiffness were posttraumatic, ranging from simple soft tissue injury (*n* = 4), fracture alone with or without heterotopic ossification (*n* = 28), fracture with dislocation (*n* = 2), dislocation alone with or without heterotopic ossification (*n* = 7) and heterotopic ossification alone (*n* = 6). The cases were treated either by oil massage or plaster of Paris cast or open reduction and internal fixation or no treatment prior to presentation[Table 1].

**Table 1:** Shows clinical description of the patients with surgical approach chosen and actual procedure/complications

Case	Duration of stiffness (months)	Diagnosis	Presurgical treatment	HO (Y/N)	Surgical approach	Procedure/complication
1	3	# Dis*: Medial capsular avulsion/post disloc	Massaging	Y	Lateral	Soft tissue procedure and excision of heterotopic ossification
2	6	Nonunion lat condyle #	K-wiring distal humerus with T condyle fracture	N	Posterior	Bony procedure - realignment and bone grafting and plating
3	3	Dis; Posterior	Native treatment	N	Posterolateral	Soft tissue procedure/vascular injury and repair
4	6	Dis; posterior	Native treatment	Y	Posterolateral	Soft tissue procedure and excision of heterotopic ossification
5	12	intercondylar # with implant *in situ*	ORIF intercondylar fracture	Y	Posterior	Soft tissue procedure, implant removal and excision of heterotopic ossification/vascular injury and repair
6	18	intercondylar # with implant *in situ*	ORIF intercondylar fracture	N	Posterior	Soft tissue release and implant removal/Neuropraxia
7	3	Soft tissue	Massaging and manipulation	N	Lateral	Soft tissue procedure
8	5	capsular avulsion #	Oil massage	N	Lateral	Soft tissue procedure
9	6	malunited intercondylar # with nonunion shaft humerus	Oil massage	Y	Lateral	Bony procedure - plating and grafting of humerus nonunion, soft tissue procedure and excision of heterotopic ossification
10	12	malunited inercondylar #	Oil massage	Y	Lateral/Medial	Bony and soft tissue procedure
11	6	coronoid chip #	Plaster of paris application	N	Lateral	Bony procedure - excision of coronoid tip - Soft tissue procedure
12	4	coronoid chip #	Plaster of paris application	N	Lateral	Bony procedure - excision of coronoid tip - Soft tissue procedure
13	8	osteochondral loose body	Plaster of paris application	N	Lateral	Bony procedure - loose body removal
14	3	radial head #	Plaster of paris application and Physiotherapy	Y	Lateral	Bony and soft tissue procedures and excision of heterotopic ossification
15	108	childhood physeal #	No treatment	N	Lateral/Medial	Bony and soft tissue procedures
16	9	Soft tissue injury	Oil massage	N	Lateral	Soft tissue procedures
17	10	lateral condyle # with malunion	Native treatment	N	Lateral	Bony and soft tissue procedures
18	24	# lateral condyle with malunion	Native treatment	N	Lateral	Bony and soft tissue procedures
19	5	HO	Oil massage	Y	Lateral/Medial	Soft tissue procedure and excision of heterotopic ossification
20	18	HO	Oil massage	Y	Lateral/Medial	Soft tissue procedure and excision of heterotopic ossification
21	12	HO: posterior disloc	Oil massage	Y	Lateral/Medial	Soft tissue procedure and excision of heterotopic ossification
22	5	HO	Native treatment	Y	Lateral/Medial	Soft tissue procedure and excision of heterotopic ossification
23	3	HO: Malunited supracondlyar #	Native treatment	Y	Lateral	Soft tissue procedure and excision of heterotopic ossification
24	4	HO: Malunited supracondylar #	Massaging	Y	Lateral/Medial	Soft tissue procedure and excision of heterotopic ossification
25	5	posterior disloc	Native treatment	N	Posterolateral	Soft tissue procedure
26	8	posterior disloc	Native treatment	N	Posterolateral	Soft tissue procedure
27	6.5	malunited intercondylar #	Plaster of paris application	N	Posterior	Bony and soft tissue procedures
28	9	Nonunion intercondylar #	Surgery, side swipe injury	N	Posterior	Bony and soft tissue procedures
29	5	Nonunion capitellum	Plaster of paris application	N	Lateral	Bony procedure - excision of loose fragment
30	7	HO; Posterior disloc	Native treatment	N	Lateral	Bony and soft tissue procedures
31	21	Monteggia # disloc	Native treatment	N	Lateral	Bony and soft tissue procedures
32	12	malunited intercondylar #	K-wiring of bicondylar fracture	N	Posterolateral	Bony and soft tissue procedures
33	18	HO	Oil massage	Y	Lateral/Medial	Bony and soft tissue procedures and excision of heterotopic ossification
34	9	HO: radial head #	Oil massage/native treatment	Y	Lateral/Medial	Bony and soft tissue procedures and excision of heterotopic ossification
35	3	HO; medial epicondyle #	Native treatment	Y	Medial	Bony and soft tissue procedures and excision of heterotopic ossification
36	4	Soft tissue	Plaster of paris application	N	Lateral	Soft tissue procedure
37	4	olecranon # with implant *in situ*	Tension band wiring olecranon-prolonged immobilization, side swipe injury	N	Lateral	Soft tissue procedure
38	7	malunited intercondylar #	Plaster of paris application	N	Lateral/Medial	Bony and soft tissue procedures
39	3.5	excision of radial head with coronoid #	Surgical excision of radial head, post surgery immobilization	N	Lateral	Soft tissue procedure
40	11	implant *in situ*-nonunion olecranon	Olecranon nonunion tension band wiring	N	Posterior	Bony procedure - Dynamic compression plating and bone grafting
41	18	Dis: posterior	Native treatment	N	Posterolateral	Soft tissue procedure
42	4	HO	Native treatment	Y	Lateral	Soft tissue procedure and excision of heterotopic ossification
43	5.5	displaced radial head#	Native treatment	N	Lateral	Bony and soft tissue procedures
44	120	Soft tissue	Oil massage/native treatment	N	Lateral	Bony and soft tissue procedures
45	7	HO	Native treatment	Y	Lateral/Medial	Soft tissue procedure and excision of heterotopic ossification
46	4.5	coronoid and radial head #	Plaster of paris application	N	Lateral	Bony and soft tissue procedures
47	8	displaced lat condyle #	Native treatment	N	Lateral	Bony and soft tissue procedures

* HO, heterotopic ossification; Y/N, yes/no; #, fracture; Dis, dislocation

Oil massaging, massaging (with available local ointments, solutions), manipulation (crudely without scientific basis), splinting with bamboo sticks are all forms of native treatment practiced locally. Word Native treatment implies mixture of all the above mentioned modalities of treatment used. Wherever specific type of native treatment was used is also mentioned

Surgery was opted when the patient had no satisfactory results after an initial trial of supervised conservative methods like range of motion (ROM) exercises, wax bath and heat therapy for three to four weeks after reporting to us. All patients were preoperatively evaluated by X-ray elbow (AP and lateral views). All patients underwent surgical release after a thorough clinical evaluation and the appropriate procedure was decided upon. Heterotopic ossification was diagnosed based on clinical assessment by local warmth, tenderness and abnormal bony mass on palpation and on radiological assessment by islands of fluffy bone within soft tissue which later ossifies into a bone mass with spurs. The three different procedures that were used in our study were soft tissue release, removal of bony blocks and/or excision of heterotopic ossification, depending on the findings as shown in Table 1.

### Operative procedure

All the patients who underwent surgical release at the elbow were exposed to regional anesthesia. The procedure that was chosen for each case was based upon the preoperative evaluation of the preexisting pathology and its complications like extensive contracture, site of heterotopic ossification, type of fracture and unreduced humeroulnar dislocation. Various approaches were used such as lateral (*n* = 23), medial (*n* = 1), posterior (*n* = 6) and posterolateral (*n* = 6) depending on the pathology. Lateral approach was preferred as it was safer and easier, but if the pathology existed in such a way that the lateral approach was not sufficient, then the medial approach was also used instead. In addition, posterolateral (*n* = 6) or posterior approach (*n* = 6) was chosen in patients with unreduced elbow dislocations and intercondylar fractures respectively, where there was stiffness in extension and the triceps lengthening was indicated.

In the lateral approach the lateral extensors were first subperiosteally elevated, thereby exposing the anterior capsule of the elbow joint. The capsule was then elevated from the lower end of the humerus, exposing the radiocapitellar joint. In order to identify the radial head, supination and pronation movements were performed. If the radial head was hypertrophied or deformed, it was excised to improve flexion. At this point, the anterior capsule was further elevated with the periosteum from the anterior aspect of the humerus until the medial border was reached. During the entire process the neurovascular bundle was protected by Hohman's retractor.

Furthermore, there is a chance that part of the coronoid process could be exposed during dissection. If the exposed region had been shown to impair flexion movement by a bony block over the coronoid fossa it was excised without much difficulty. Then dissection of the posterior aspect of the humerus to free the capsular attachment was done subperiosteally. In order to improve extension, the olecranon fossa was cleared of all fibrous tissue. Sometimes the tip of the olecranon process was excised up to 50% and yet the triceps attachment remained intact. This excision resulted in a greater extension arc to the elbow. However, it must be noted that if the soft tissue release is inadequate, then a bony block must be looked for or else the second approach, the medial approach, is added up for sequential release (*n* = 11). In this process, the ulnar nerve was first isolated and protected. Next, the flexor origins were subperiosteally elevated and the anterior capsule with the periosteum was stripped from the lower end of the humerus, thereby giving a better view of the elbow joint. In order to attain maximum movements of the elbow, further inspection of the humeroulnar joint was done to find any bony blocks. Any extraarticular ossified mass in the front was excised with care, as the neurovascular bundle was nearby. If ever there was any doubt, the release of the tourniquet helped to identify the brachial artery at least once or twice during surgery, thereby reducing the risk of any damage. In addition, the exuberant callus surrounding the bony margins was excised in order to attain the normal contour of the bone. However, it is mandatory to preserve the medial collateral ligament to preserve the stability of the joints.

After the soft tissue release, the bony block was excised. Then assessment of the flexion was carried out; if the triceps muscle was tight and flexion more than 90° was not possible, V-Y plasty of triceps was carried out (*n* = 7).

Finally, flexion and extension of the elbow were carried out passively to evaluate the gain in movement. The pneumatic tourniquet was released and hemostasis was obtained. After surgery, the wound was closed with suction drainage.

### Postoperative protocol

The patients were immobilized in long arm crammer wire splint in 90° of flexion to keep the wound without much tension on suture site. The patients were asked to perform gentle active movements under the supervision of the physiotherapist within tolerable limits of pain in accordance with the wound healing for the first 10 days. The splint was removed during the exercise. Active exercises were carried out for the neighboring joints as well to maintain the range of motion. All the patients were given a fixed dose of 25 mg of Indomethacin twice daily for a period of six weeks to prevent heterotopic ossification^9^ (Indomethacin is contraindicated in pediatric age, < 12 years). Patients were followed every 10 days for the first two months, followed by monthly thereafter. Patients were followed in the physiotherapy department daily for the first six weeks, monthly thereafter.

After the wound healing, patients were encouraged to increase the active exercises. The supportive therapy like ice packs was given for the first six weeks. Also, as time progressed the patients were given tubular compression bandage to provide support and to reduce the swelling in the released elbow. Three weeks after surgery, the splint was totally discarded and the patients were encouraged to do vigorous active movements that would help in increasing the range of motion. All the measurements were recorded using a goniometer.

## RESULTS

**Figure 1:**
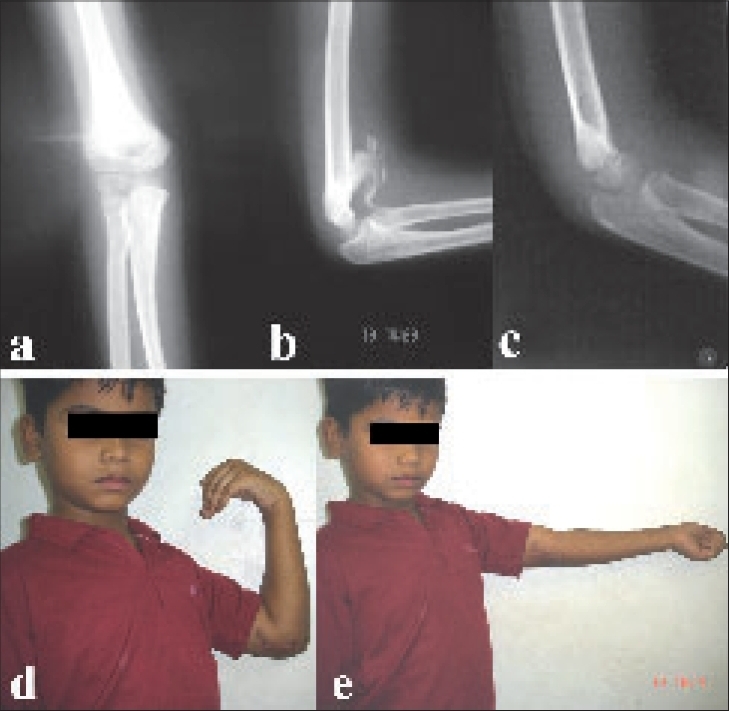
Preoperative anteroposterior (a) and lateral radiographs (b) of right elbow in 13 Year old male showing anterior heterotopic bone causing limitation of flexion. Postoperative lateral radiographs (c) following arthrolysis and removal of anterior bony block. Clinical photographs (d and e) showing a good range of flexion and extension at follow up.

In our study, all 47 patients gained good range of motion [Table 2] following surgical release [Figure 1]. The mean preoperative Mayo Clinic Performance score [Table 3] was 66.59 and the mean postoperative Mayo Clinic Performance score was 93.82, with a mean improvement of 27.23 (‘*t*’ test value was 14.48, *P* < 0.01). The mean (and standard deviation) preoperative range of motion was 33.9° (±24.0) and the mean (and standard deviation) postoperative range of motion at the time of last follow-up was 105° (±19.47), indicating a mean gain of 71.1° in the range of motion (*t* ` test value is 19.27 which is significant at 0.01 level, *P* < 0.01). None of the patients ever had any symptomatic instability with sequential release which needed further procedure, though objective Mayo's Assessment Score showed four cases of moderate instability. None of these patients had pain, apprehension or subluxation.

**Table 2:** Results

Case	Preop ROM* (degree)	Postop ROM* (degree)	Preop arc (degree)	Postop arc (degree)	Improvement ROM* arc	MAYO score(10 P*/M*/ADL*/I* (45/20/25/10)
1	80-100	5-110	20	105	85	100 45/20/25/10
2	90-100	30-120	10	90	80	100 45/20/25/10
3	0 fixed extension	20-120	0	100	100	95 45/20/25/5
4	0 fixed extension	20-125	0	105	105	100 45/20/25/10
5	80-90	30-130	10	100	90	95 45/20/20/10
6	40-80	20-130	40	110	70	95 45/20/20/10
7	90-100	0-110	10	110	100	75 30/15/20/10
8	70-100	30-130	30	100	70	80 30/20/20/10
9	40-70	40-100	30	60	30	65 45/5/5/10
10	50-110	20-120	60	100	40	100 45/20/25/10
11	10-110	0-135	100	135	35	100 45/20/25/10
12	20-115	10-125	95	115	20	100 45/20/25/10
13	30-120	0-130	90	130	40	100 45/20/25/10
14	60-90	15-125	30	110	80	100 45/20/25/10
15	90-100	60-120	10	60	50	95 45/20/25/10
16	50-90	20-130	40	110	70	95 45/20/20/10
17	50-90	10-140	40	130	90	100 45/20/25/10
18	80-100	20-130	20	110	90	95 45/20/20/10
19	30-40	20-120	10	100	90	95 45/20/20/10
20	40-90	20-135	50	115	65	100 45/20/25/10
21	50-80	20-100	30	80	50	100 45/20/25/10
22	45-100	20-130	55	110	55	100 45/20/25/10
23	60-90	20-140	30	120	90	100 45/20/25/10
24	80-120	10-140	40	130	90	100 45/20/25/10
25	90-100	10-115	10	105	95	85 30/20/25/10
26	70-80	10-120	10	110	100	100 45/20/25/10
27	80-110	20-130	30	110	80	100 45/20/25/10
28	70-90	30-120	20	90	70	95 45/20/20/10
29	80-110	10-130	30	120	90	85 30/20/25/10
30	60-90	20-140	30	120	90	80 30/20/25/5
31	80-110	10-120	30	110	80	100 45/20/25/10
32	70-100	60-110	30	50	20	75 45/15/5/10
33	80-110	10-140	30	130	100	100 45/20/25/10
34	20-90	10-110	70	100	30	100 45/20/25/10
35	60-115	30-130	55	100	45	95 45/20/20/10
36	80-110	10-120	30	110	80	100 45/20/25/10
37	60-110	10-130	50	120	70	100 45/20/25/10
38	80-100	50-120	20	70	50	75 45/15/15/10
39	70-90	10-135	20	125	105	100 45/20/25/10
40	60-90	20-100	30	80	50	95 45/15/25/10
41	60-80	30-120	20	90	70	90 45/15/20/10
42	45-80	0-140	35	140	105	95 45/20/25/5
43	60-90	15-120	30	105	75	100 45/20/25/10
44	20-110	20-120	90	100	10	80 30/20/25/5
45	45-90	10-135	45	125	80	100 45/20/25/10
46	70-90	10-110	20	100	80	100 45/20/25/10
47	60-70	30-120	10	90	80	75 30/20/20/5

ROM, range of motion: P. pain; movements; ADL, activities of daily living: I, instability

**Table 3:** Mayo clinic performance score 19

Category	Description	Max. points
Pain (max, 45 points)	None	45
	Mild	30
	Moderate	15
	Severe	0
Range of motion	Aro < 100°	20
(max, 20 points)	Arc 50-1 00°	15
	Arc > 50°	5
Function (max, 25 points)	Able to comb hair	5
	Able to feed oneself	5
	Able to perform	5
	personal hygiene tasks	5
	Able to put on shirt	5
	Able to put on shoes	
Stability (max, 10 points)	Stable	10
	Moderately unstable	5
	Grossly unstable	0
Total 100 points		

In patients who underwent surgical release from three to six months (*n* = 24) following the injury, the mean improvement in range of motion was 75.5° and patients who had surgery delayed for more than six months (*n* = 23) had a mean gain of 61.9° in the range of motions. Hence, patients who had undergone surgery without a prolonged waiting period had better results compared to patients in whom surgical release was delayed.

When the type of injury causing the stiffness was taken into account, patients having dislocation of elbow alone (*n* = 7) exhibited better gain in the range of motion (86.7°), irrespective of time i.e. either early or late surgical release, when compared to other injuries like fracture dislocation (*n* = 2) (85° range of motion), fracture (*n* = 28) (64.6°) and soft tissue injuries (*n* = 4) (66°).

The majority of the patients had received some type of prior treatment before undergoing surgery for elbow stiffness. Those individuals who had received native treatment (*n* = 29) (oil bandage massaging) prior to surgical release showed a mean benefit of 75.2° range of motion (ROM). Patients who had an elbow corrective surgery and fracture fixation (*n* = 8) resulting in stiffness showed a mean improvement of 69.4° (ROM) after the release. The individuals who had applied a plaster of Paris (*n* = 9) resulting in stiffness showed a mean improvement of 61.7°.

Comparing the patients according to the age group, patients below the age of 25 years (*n* = 24) had 70.5° mean gain in range of motion. Patients above 25 years (*n* = 23) had 67.6° mean gain in range of motion indicating that good results are obtained in younger age group than in the older age group.

Basically there were three groups: (1) Stiffness with associated ununited/malunited fracture or dislocation, (2) Stiffness without heterotopic ossification and (3) Stiffness with heterotopic ossification.

**Figure 2:**
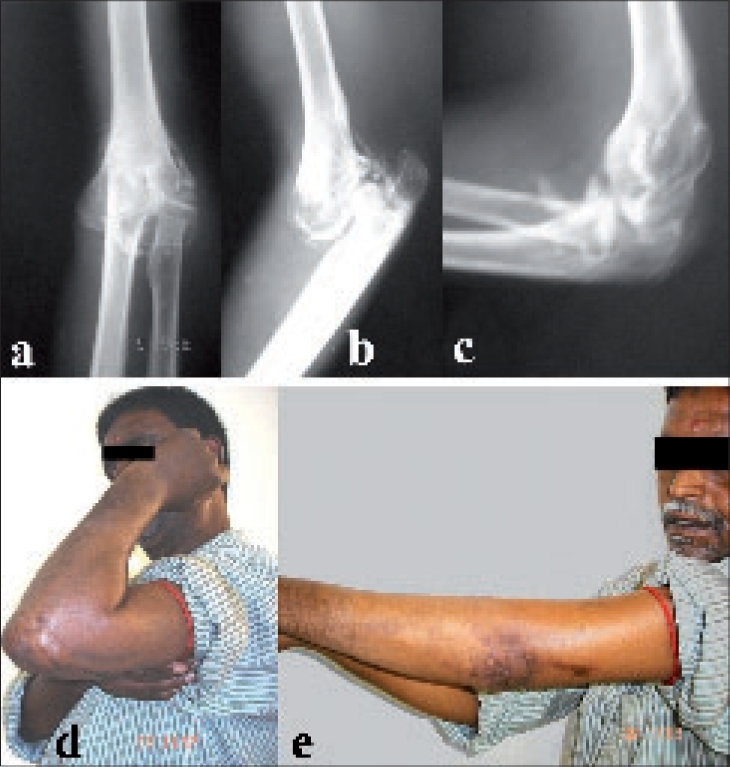
Anteroposterior (a) and lateral radiographs (b) of unreduced posterior dislocation of left elbow with myositis ossificans in an adult male. Postoperative lateral radiograph (c) showing reduced elbow with good range of motion with excised myositis ossificans. Clinical photographs (d and e) showing postoperative range of motion at two years of follow-up

Thirty-seven patients had stiffness due to fracture or dislocation and after surgical treatment had mean gain in range of motion of 69.8°. Six patients had posttraumatic stiffness with heterotopic ossification (fig 2) and after surgical treatment had mean gain in range of motion of 82.5°. Four patients had posttraumatic stiffness without heterotopic ossification and after surgical treatment had mean gain in range of motion of 65°. Comparing the patients in whom the normal bony contour was maintained (*n* = 31) with the patients not having normal bony contour (*n* = 16), the mean gain in range of motion was 75.8° and 61.9° respectively indicating that good results are seen in patients when the bony contour is maintained. The delineation between abnormal and normal bony contour was made based on preoperative radiological and per-operative findings. Similarly, patients having humeroulnar articular cartilage damage, as assessed during surgery, with mean gain in range of motion of 66.5°, faired poorly compared with patients not having humeroulnar articular damage who had mean gain in range of motion of 75.9°.

Out of 47 cases of elbow stiffness, a large number of cases (17 patients) had heterotopic ossification. When patients having heterotopic ossification were compared with the patients not having heterotopic ossification, the results were 72.4° mean gain in range of motion and 70.3° mean gain in range of motion respectively.

Patients who did not have heterotopic ossification and underwent surgery from three to six months post injury had a mean gain of 73.5°. In patients who waited for more than six months had mean gain of 66.8°. In cases having heterotopic ossification where release was performed from three months to six months had net gain of 77.5° in range of motion (Figure 2a-e). Cases in which release was performed after six months had mean gain of 57.1°. Thus, in the heterotopic ossification group surgical release at the earliest yielded better results than delayed cases as suggested by Park *et al.*^10^ Our study is a medium term result on elbow arthrolysis so we are unable to comment on the maintenance of range of motion with time^11^–^13^ though there is no fall in the score in patients who had been followed for five years. Comparing the type of treatment that the patients had undergone prior to the surgical release, in patients with heterotopic ossification (*n* = 17), we found that patients who had native treatment had higher incidence (*n* = 15), and the incidence was less in other treatments like plaster of Paris cast (*n* = 1) and previous surgery (*n* = 1).

After assessing the patients on the basis of improvements in range of motion a different type of measurement *“Mayo Clinic Performance Score”* was used, that scored the patients on the basis of pain, motion, stability and activities of daily living. Out of 47 patients, 25 patients scored a perfect score of 100 on this test which indicated an excellent result; in fact the average score was 95.53 out of 100.

The paired sample ‘*t*’ test was used to test significant difference between preoperative arc and postoperative arc. The ‘*t* ’ test value is found to be 19.27 which is significant at 0.01 level (*P* < 0.01).

## DISCUSSION

Most of the patients who reported with posttraumatic stiff elbow were due to inappropriate primary management like inadequate primary fixation, native treatment with tight bandages, oil massaging and forceful manipulation of the elbow. Forceful manipulations through passive movements work against the natural healing process leading to heterotopic ossification. All the cases were treated with sequential release if there was no progress after initial conservative treatment, with minimum of four weeks of active physiotherapy and assessment from the time of presentation, except in unreduced dislocations.

One major concern initially was excising heterotopic bone within such a short period of time before maturation. All the patients were operated after a trial of conservative management at the time of presentation without a prolonged waiting period for maturity of heterotopic bone^4^ from three months post injury.

After performing surgical release in 47 patients we found that those who underwent excision of heterotopic bone within a period of six months gained a better range of motion, than those who waited for more than six months Previous recommendation that one should wait 12-18 months before removal of heterotopic bone presently holds good in patients with head injury, burns and polytrauma because of continuing heterotopic ossification.^14^ In posttraumatic non-neurogenic etiology there is no recurrence after early excision^15^–^17^ hence we need not wait for maturity of heterotopic ossification before excision. None of the patients had aggravating systemic illness like burns, spinal cord injury and head injury and no one had increased heterotopic ossification following surgery.

We support research promoting early excision of heterotopic bone, as it yielded the maximum gain in movement at the elbow although, there is contrary view available.^18^ Our study also confirms that longer the elbow remains stiff, poorer the prognosis.

We used lateral, medial, posterior and posterolateral approach. Lateral approach was the most often used and this provided the greatest gain in range of motion at the elbow without instability. The study performed by Mansat and Morrey^3^ used exclusively the lateral approach with desirable results. The medial approach was not used as much as the lateral approach. We used the medial approach only if there was a need for fracture fixation in this region or further release was necessary to gain movement. We still found the medial approach to produce good results as all patients had a gain in range of motion. Our results are comparable with other studies that have focused on surgical release of a stiff elbow using the medial approach. Wada, Ishii, Usui and Miyano^18^ reported significantly improved the range of motion at the elbow with the use of medial approach for operative release following trauma. They obtained a mean increase of 64° in the arc of motion of their patients whereas we obtained a mean increase of 89.5°. Both these studies show that the medial and lateral approaches produce good results. We feel that it is imperative to address the pathology causing stiffness with a preoperative planning of the approach, to get good results. In order to attain maximum flexion and extension one or more of the approaches may be necessary to gain access to all of the structures.

In the immediate postoperative period patients were encouraged on active range of motion under supervision of physiotherapist except in unstable situation like open reduction of chronic elbow dislocation where initial immobilization for three weeks followed by active range of motion exercises were started.

We had two cases of vascular injury. The first case was due to a per-operative direct vascular injury which was recognized immediately. The second case had a delayed presentation, which occurred six hours after the time of surgery due to an initial intimal tear and late thrombosis. Both patients had immediate vascular repair; however, they had neuropraxia, which recovered fully by six weeks. We have changed our practice since then by releasing the tourniquet at least twice in between the procedure to check the circulation. There were no cases of reflex sympathetic dystrophy or resurgery except for vascular repair.

The limitation of our study is that it did not include pediatric cases (age < 12 years), infected elbow cases, preoperative CT evaluation for Heterotopic ossification (HO) and the results were not compared with the arthroscopic elbow arthrolysis. We have not compared any of our groups because of a small sample size and a lot of variables; we have only analyzed the results of individual groups and their end result.

In summary, elbow arthrolysis is a good procedure which gives a useful gain in range of motion provided one is wary of vascular injury. This systematic approach and method seemed to work out well confirming the validity of our approach. We also conclude that it is most beneficial to excise heterotopic ossification in the first six months following injury. We recommend this procedure in treatment of posttraumatic elbow stiffness as it restores normal elbow function.
